# Regulation of breast cancer induced bone disease by cancer-specific IKKβ

**DOI:** 10.18632/oncotarget.24743

**Published:** 2018-03-23

**Authors:** Silvia Marino, Ryan T. Bishop, Mattia Capulli, Antonia Sophocleous, John G Logan, Patrick Mollat, Barbara Mognetti, Luca Ventura, Andrew H. Sims, Nadia Rucci, Stuart H. Ralston, Aymen I. Idris

**Affiliations:** ^1^ Department of Oncology and Metabolism, University of Sheffield, Sheffield, UK; ^2^ Bone and Cancer Group, Edinburgh Cancer Research Centre, University of Edinburgh, Western General Hospital, Edinburgh, UK; ^3^ Rheumatology and Bone Disease Unit, Centre for Genomic and Experimental Medicine, University of Edinburgh, Western General Hospital, Edinburgh, UK; ^4^ University of Turin, Department of Clinical and Biological Sciences, Orbassano, Italy; ^5^ Galapagos SASU, Romainville, France; ^6^ University of L’Aquila, Department of Biotechnological and Applied Clinical Sciences, L’Aquila, Italy; ^7^ Department of Pathology, San Salvatore Hospital, L’Aquila, Italy; ^8^ Applied Bioinformatics of Cancer, MRC Institute of Genetics and Molecular Medicine, University of Edinburgh, Western General Hospital, Edinburgh, UK

**Keywords:** IKKβ, NFκB, breast cancer, bone metastasis, osteolysis

## Abstract

NFκB is implicated in breast cancer bone metastasis and skeletal remodelling. However, the role of IKKβ, a key component of the canonical NFκB pathway, in the regulation of breast cancer osteolytic metastasis has not been investigated. Here, we describe the cancer-specific contribution of IKKβ to bone metastasis, skeletal tumour growth and osteolysis associated with breast cancer. IKKβ is highly expressed in invasive breast tumours and its level of expression was higher in patients with bone metastasis. IKKβ overexpression in parental MDA-MD-231 breast cancer cells, promoted mammary tumour growth but failed to convey osteolytic potential to these cells in mice. In contrast, IKKβ overexpression in osteotropic sub-clones of MDA-MB-231 cells with differing osteolytic phenotypes increased incidence of bone metastasis, exacerbated osteolysis and enhanced skeletal tumour growth, whereas its knockdown was inhibitory. Functional and mechanistic studies revealed that IKKβ enhanced the ability of osteotropic MDA-MB-231 cells to migrate, increase osteoclastogenesis, and to inhibit osteoblast differentiation via a mechanism mediated, at least in part, by cytoplasmic sequestering of FoxO3a and VEGFA production. Thus, tumour-selective manipulation of IKKβ and its interaction with FoxO3a may represent a novel strategy to reduce the development of secondary breast cancer in the skeleton.

## INTRODUCTION

Bone metastases and skeletal related events in patients with metastatic breast cancer are an important cause of morbidity, which place significant demands on health care resources [1, 2]. Clinical studies have shown that approximately 70% of breast cancer patients with advanced disease develop lytic bone lesions and suffer from skeletal-related events [1, 3, 4]. Breast cancer osteolytic metastasis develops and expands because of an interaction between tumour cells and local cells in the tumour microenvironment such as osteoclasts, osteoblasts and their precursors [1, 2]. Bone resorption by osteoclasts elicits the release of factors from osteoblasts and bone matrix that in turn stimulate tumour growth, exacerbate osteolysis and cause bone loss [1, 2]. Many tumour- and bone-derived factors have been implicated in the pathogenesis of bone metastases and osteolysis associated with breast cancer [4, 5]. However so far very few therapeutic targets have been identified.

The nuclear factor-κB (NFκB) signaling pathway plays an important role in breast cancer progression, inflammation and bone cell activity [6]. The IκB kinase (IKK) complex, a key component of the canonical NFκB signalling pathway, is comprised of two closely related catalytic subunits, IKKα and IKKβ and a regulatory subunit IKKγ [6]. Ligand-induced activation of IKK triggers the phosphorylation and recruitment of IKKα, IKKβ and IKKγ. Of the two catalytic subunits, IKKβ has emerged as the main regulator of osteoclast and osteoblast differentiation. Preclinical studies in mouse models of inflammation-induced bone loss and postmenopausal osteoporosis have shown that genetic inactivation or pharmacological inhibition of the IKKβ/NFκB signalling in bone cells inhibits osteoclastic bone resorption [7–10] and promotes bone formation [11, 12].

IKKβ is also implicated in breast cancer tumorigenesis and metastasis. High levels of expression and activity of IKKβ correlate with drug and radiotherapy resistance and poor clinical outcome in breast cancer patients [13–16]. A plethora of tumour-derived factors have been shown to regulate breast cancer tumorigenesis through activation of the IKKβ/NFκB signalling pathway [17–21]. Furthermore, accumulating recent evidence suggests that a host of oncogenic and cancer-driver pathways including c-Myc, Forkhead box O3A (FoxO3a) and PI3/Akt/mTOR regulate breast cancer cell metastatic behaviour through interaction with IKKβ [17–21].

There has been an increasing interest in the therapeutic targeting of the IKK/NFκB signalling pathway for the treatment of cancer associated bone disease. Both the canonical and non-canonical NFκB signalling pathways play a role in breast cancer bone metastasis [10], and we have previously reported that the small-molecule inhibitors of these pathways, Celastrol and Parthenolide, reduced the development of osteolysis in a rat model of breast cancer-induced osteolysis [22]. However, the role of IKKβ, a key unit of the canonical NFκB signalling pathway, in the regulation of bone metastasis, skeletal tumour growth and osteolysis has not been investigated. Here, we report that IKKβ in sub-clones of the human MDA-MB-231 breast cancer cells with differing osteolytic phenotypes plays an essential role in bone metastasis, skeletal tumour burden and osteolysis associated with breast cancer.

## RESULTS

### IKKβ expression is enhanced in breast cancer bone metastasis

**Figure 1:**
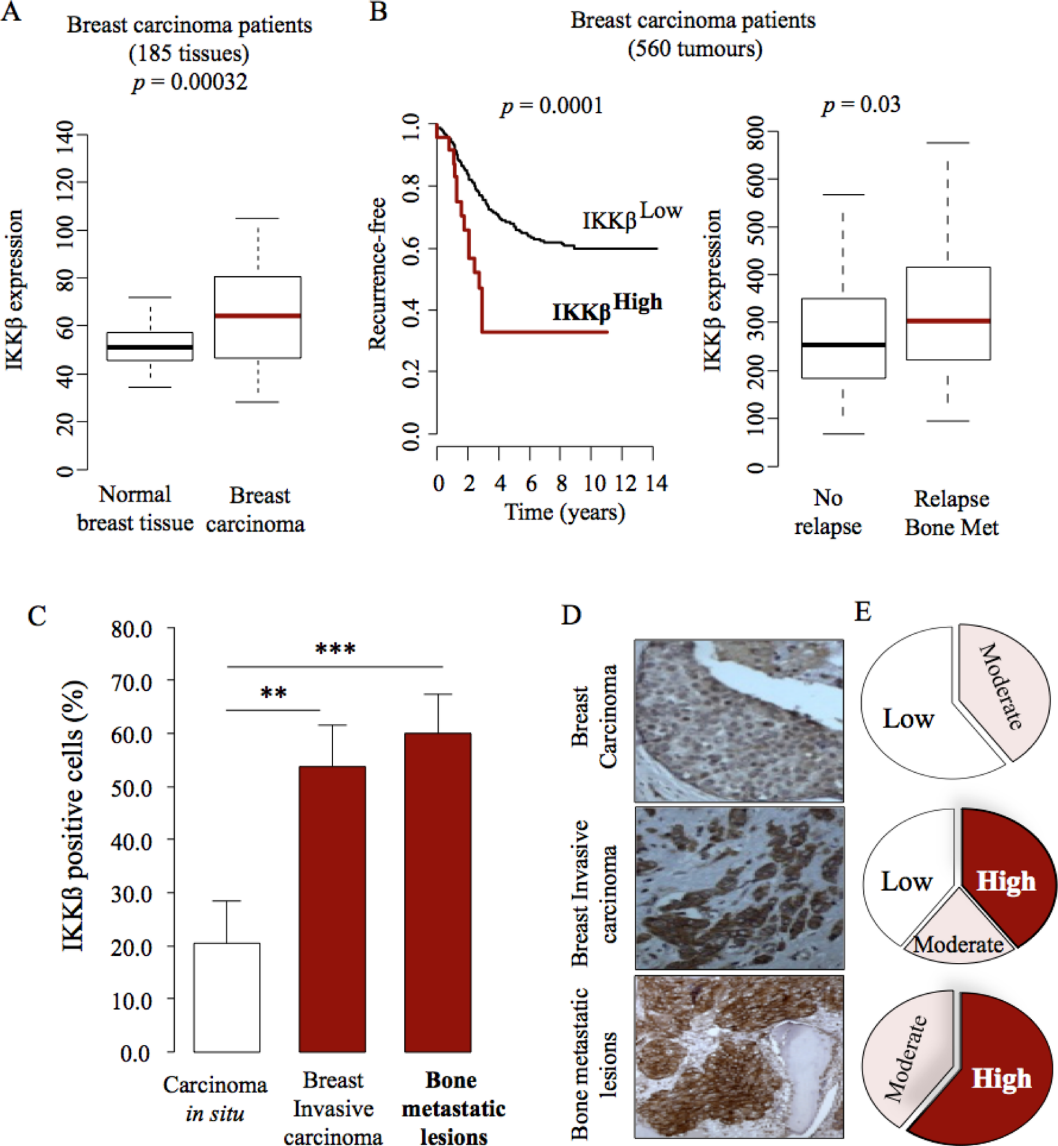
IKKβ expression is associated with high risk for bone metastasis (**A**) Retrospective analysis of breast tissue samples showing increased expression of IKKβ in breast carcinoma when compared to normal breast tissue (*n* = 185: 143 normal and 42 invasive ductal carcinoma). (**B**) Increased expression of IKKβ is associated with breast cancer recurrence (left panel) and bone metastasis relapse (right panel) in patients (*n* = 560: 185, bone metastasis, 375, no bone relapse). Expression of IKKβ in the experiment described in panels A to B was determined by mRNA analysis. The most significant cut-point for high/low-IKKβ is shown (left panel). (**C**) IKKβ expression is increased in primary invasive carcinomas and bone metastatic lesions in a clinical dataset of matched primary tumour (*n* = 18) and bone biopsies (*n* = 5) from a breast cancer patient cohort. Expression of IKKβ in the experiment described in panel C was determined by Immunohistochemistry. Values are mean ± SD; ^**^p < 0.01 and ^***^p < 0.001. (**D**–**E**) Representative photomicrographs (D) and Pie chart (E) show IKKβ expression in tumour biopsies and bone sections from patient #3102/07 from the breast cancer patient cohort described in C (right panel, black). the percentage of IKKβ positive cells that ranges from approximately 80% (bone metastasis) to 20% (normal tissue) of total tissue area. Values are mean ± SD.

The canonical NFκB signaling pathway promotes breast cancer bone metastasis [10, 22]. Given that IKKβ is a key component of this pathway, we first assessed its expression in clinical datasets of human primary breast carcinoma and bone biopsies from breast cancer patients with known clinical outcome (Figure 1). A retrospective analysis of a cohort of patients with metastatic breast cancer [23] revealed that mRNA expression of IKKβ is elevated in primary breast carcinoma (Figure 1A, *p* = 0.00032, *n* = 185) and it is strongly associated with disease recurrence (Figure 1B, left panel, *p* = 0.0001, *n* = 560) and bone metastasis relapse (Figure 1B, right panel, *p* = 0.03, *n* = 560). To further evaluate the clinical importance and potential role of IKKβ in breast cancer bone metastasis, we performed detailed immunohistochemical assessment of IKKβ expression in breast tumours and lytic lesions in bone in biopsies from breast cancer patients who developed bone metastasis. As shown in Figure 1 (panels C–E), expression of IKKβ is clearly evident in breast carcinoma *in situ* (Figure 1C) and its level of expression were increased in invasive breast carcinomas (Figure 1C) and in lytic lesions (Figure 1C) when compared to breast carcinomas. Representative photomicrographs and Pie chart that show IKKβ expression in tumour biopsies and bone sections from breast cancer patient #3102/07 are shown in Figure 1, panels D and E, respectively. These results together indicate that cancer-specific expression of IKKβ is implicated in breast cancer bone metastasis.

### Cancer-specific IKKβ enhances mammary tumour growth

**Figure 2:**
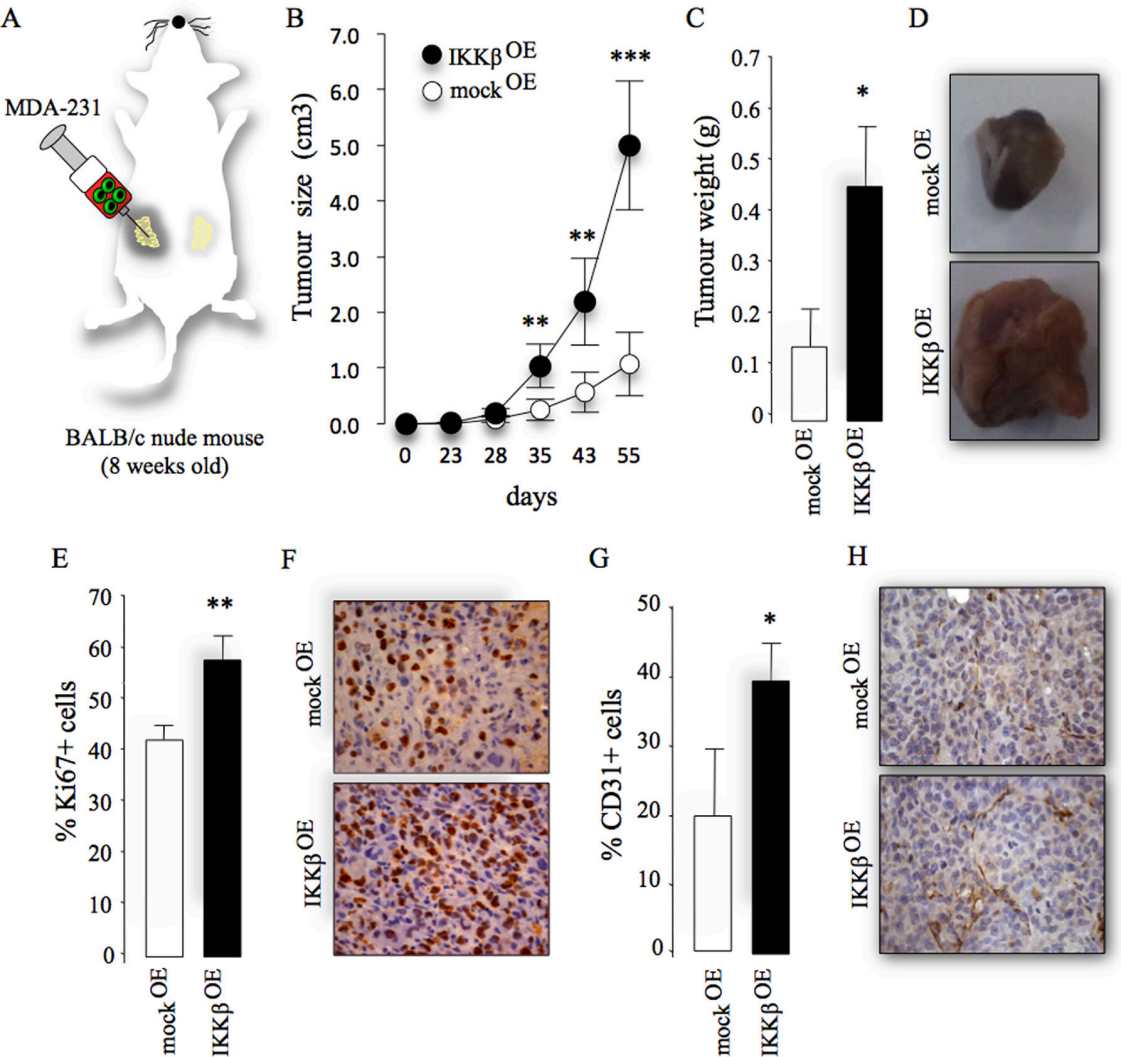
Cancer-specific IKKβ enhances primary tumour growth (**A**) Graphic representation of orthotopic injection of parental human MDA-231 overexpressing IKKβ cells (IKKβ^OE^) or their control (mock^OE^) into the mammary fat pads of adult mice. (**B**–**C**) Tumour size (cm^3^, B) and weight (gram, C) from the experiment described in panel A. (**D**) Representative photomicrographs of tumours from the experiment described. *E)* Percentage of Ki67 positive cells in tumour biopsies from the experiment described. (**F**) Representative photomicrographs of histological sections of tumours showing Ki67 from the experiment described. (**G**) Percentage of CD31 positive cells in tumour biopsies from the experiment described. (**H**) Representative photomicrographs of histological sections of tumour biopsies showing CD31 positive cells from the experiment described. Values are mean ± SD; ^*^p < 0.05; ^**^p < 0.01; ^***^p < 0.001.

Metastatic breast cancer cells accumulate oncogenic alterations that affect their ability to metastasise to bone [24, 25]. With this in mind, we assessed the protein level of IKKβ in different clones of the triple-negative human MDA-MB-231 (MDA-231) and mouse 4T1 cells and their sub-clones with different propensity to metastasise to and colonize the skeleton. This experiment confirmed that IKKβ expression is significantly higher in the osteotropic human MDA-231-BT1 (moderate) and MDA-231-BT2 (aggressive) (Supplementary Figure 1A), and mouse 4T1-BT1 cells (aggressive) (Supplementary Figure 1B), when compared to their parental controls. In view of IKKβ being highly expressed in the osteotropic sub-clones of human MDA-231 breast cancer cells, we hypothesized that over-expression of IKKβ in parental MDA-231 cells conveys the capability to these cells to metastasise to bone from the mammary fat pads. Human MDA-231 breast cancer cells were chosen as a model of breast cancer bone metastasis in this study because the parental sub-clone of these cells has not previously been reported to metastasize to bone in mice after orthotopic injection in the mammary fat pad (unlike the 4T1 cells; Idris *et al.* unpublished data). Stable over-expression of IKKβ (Supplementary Figure 1C) significantly enhanced tumour growth of parental MDA-231 cells after orthotopic injection (Figure 2A), as evidenced by increased tumour size (Figure 2B and Supplementary Figure 2A) and tumour weight (Figure 2C–2D). Ki67 and CD31 staining of histological tissue sections revealed a greater number of proliferating cells (Figure 2E–2F) and CD31 positive vessels (Figure 2G–2H) in tumour biopsies from mice injected with MDA-231 cells over-expressing IKKβ, indicative of enhanced tumour growth and angiogenesis, respectively. However, contrary to our hypothesis, IKKβ overexpression failed to convey metastatic capability to the parental MDA-231 from the mammary fat pad to bone in the model described.

### Cancer-specific IKKβ exacerbates osteotropic breast cancer cell metastasis to bone

**Figure 3:**
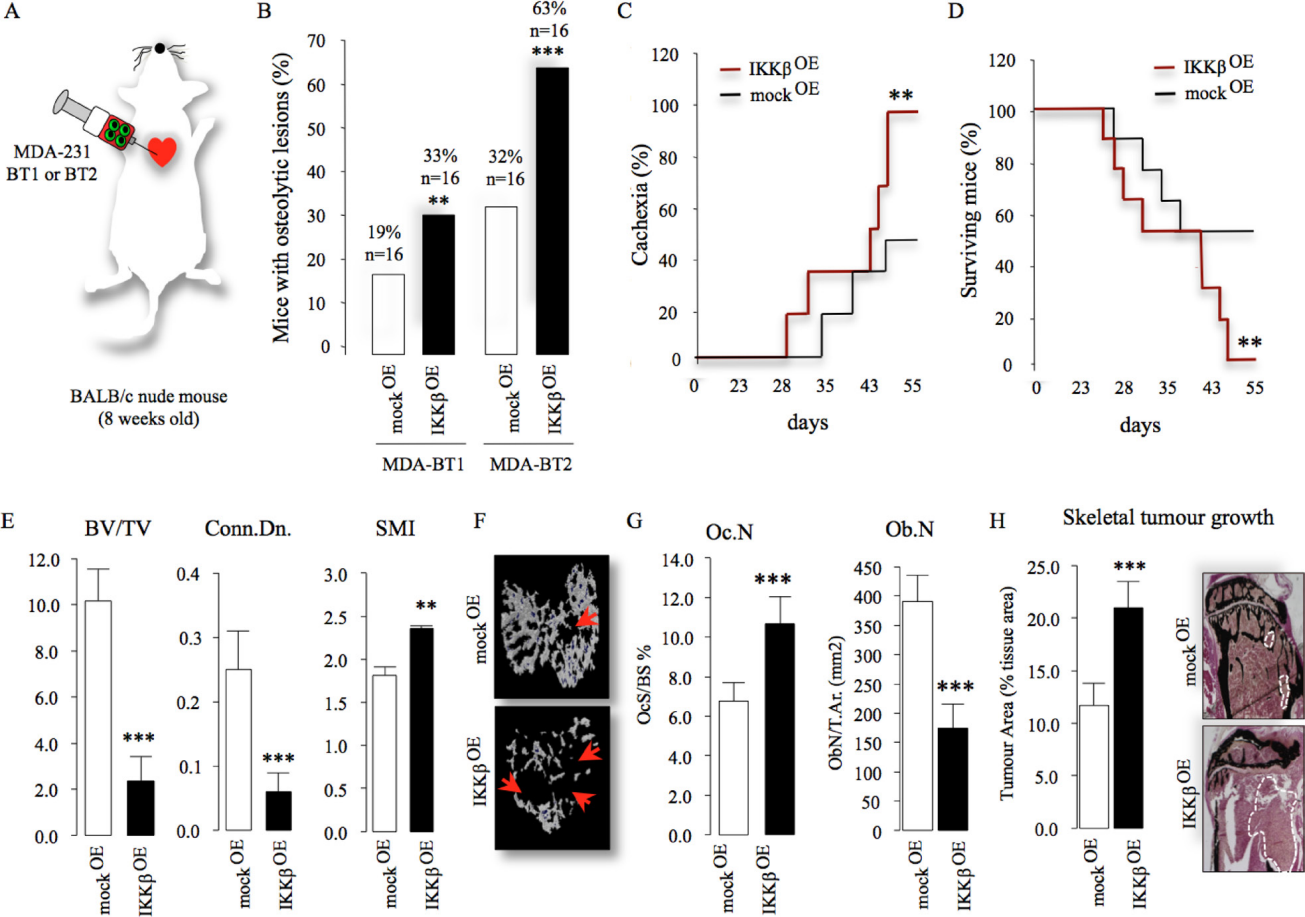
Cancer-specific IKKβ exacerbates bone metastasis and enhances skeletal tumour growth (**A**) Graphic representation of intra-cardiac injection of the osteotropic MDA-231-BT1 (MDA-BT1, *n* = 16) and MDA-231-BT2 (MDA-BT2, *n* = 16) (55 days) overexpressing IKKβ (IKKβ^OE^) or their control (mock^OE^) in adult BALB/c -nu/nu athymic mice. (**B**) Percentage of osteolytic metastasis incidence from two independent populations of osteotropic human MDA-231 breast cancer cell-lines (*n* = 16/group) from the experiment described in panel A as assessed by x-ray and microCT. (**C**–**D**) Percentage of cachexia (C) and survival (D) in mice after intra-cardiac injections of the osteotropic MDA-231-BT2 (*n* = 16) (55 days) overexpressing (IKKβ^OE^) or their control (mock^OE^). (**E**) Bone volume (BV/TV, %, left) and trabecular connectivity (Conn.Dn., middle and SMI, right) after intra-cardiac injections of the MDA-231-BT2 overexpressing (IKKβ^OE^) or their control (mock^OE^) in adult BALB/c mice (*n* = 16) (up to 55 days). (**F**) Representative photomicrographs of microCT scan of tibial metaphysis of mice from the experiment described in panel E. Arrowheads denote osteolysis bone damage. (**G**) *In vivo* osteoclastogenesis (Oc.N, left panel) and number of osteoblasts (Ob.N, right panel) from tibial metaphysis of adult BALB/c mice from the experiment described in panel E (*n* = 16). H) Tumour area (% of tissue area) in tibial metaphysis of mice from the experiment described in panel C–G (*n* = 5, mock^OE^ and *n* = 7, IKKβ^OE^). Representative photomicrographs of tumour in tibial metaphysis of mice from the experiment described are shown in the left panel. Dotted line denotes tumour. Values are mean ± SD; ^**^p < 0.01; ^***^p < 0.001.

Subsequently, we went to show that IKKβ overexpression enhanced the ability of two osteoporotic sub-clones of MDA-231 cells to cause bone metastasis in mice after intra-cardiac injection [26] (Figure 3A). Micro-computed tomography (MicroCT) and x-ray analysis revealed that all mice that received intra-cardiac injection of the osteotropic MDA-231-BT1 (moderately metastatic) and MDA-231-BT2 (highly metastatic) exhibited increased number of lytic lesions (MDA-231-BT1, 68% increase and MDA-231-BT2, 90% increase, *p <* 0.05) (Figure 3B) compared to control mice. Furthermore, mice injected with the moderately metastatic sub-line MDA-231-BT1 overexpressing IKKβ experienced severe cachexia (Figure 3C, 40% increase after 55 days, *p <* 0.05) and became morbid and were sacrificed before 55 days compared to only 50% from the mock control group (*p <* 0.05) (Figure 3D). Of note, all mice injected with human breast cancer cells over-expressing IKKβ exhibited evidence of metastases to the lungs when compared to mock control (data not shown). Detailed MicroCT of the tibial metaphysis of the sacrificed mice injected with human MDA-231-BT2 (aggressive) cells overexpressing IKKβ at the end of the experiment showed a significant reduction in bone volume (Figure 3E, left and 3F), trabecular connectivity (decrease in connectivity density, Figure 3E, middle) and a more rod-like appearance of the trabeculae (increased structure model index, Figure 3E, right) compared to mock control. A detailed bone histomorphometric analysis of histological samples revealed that mice injected with the osteotropic MDA-231-BT1 cells overexpressing IKKβ exhibited dramatic increase in osteoclast number (Figure 3G, left) and significant inhibition in osteoblast number (Figure 3G, right). Furthermore, IKKβ overexpression increased skeletal tumour growth when compared to the control group (Figure 3H). Collectively, these results demonstrate that cancer-specific IKKβ plays an essential role in the regulation of bone metastasis, skeletal tumour burden and osteolysis in the mouse model of human breast cancer described.

### Cancer-specific IKKβ regulates breast cancer induced bone cell activity

**Figure 4:**
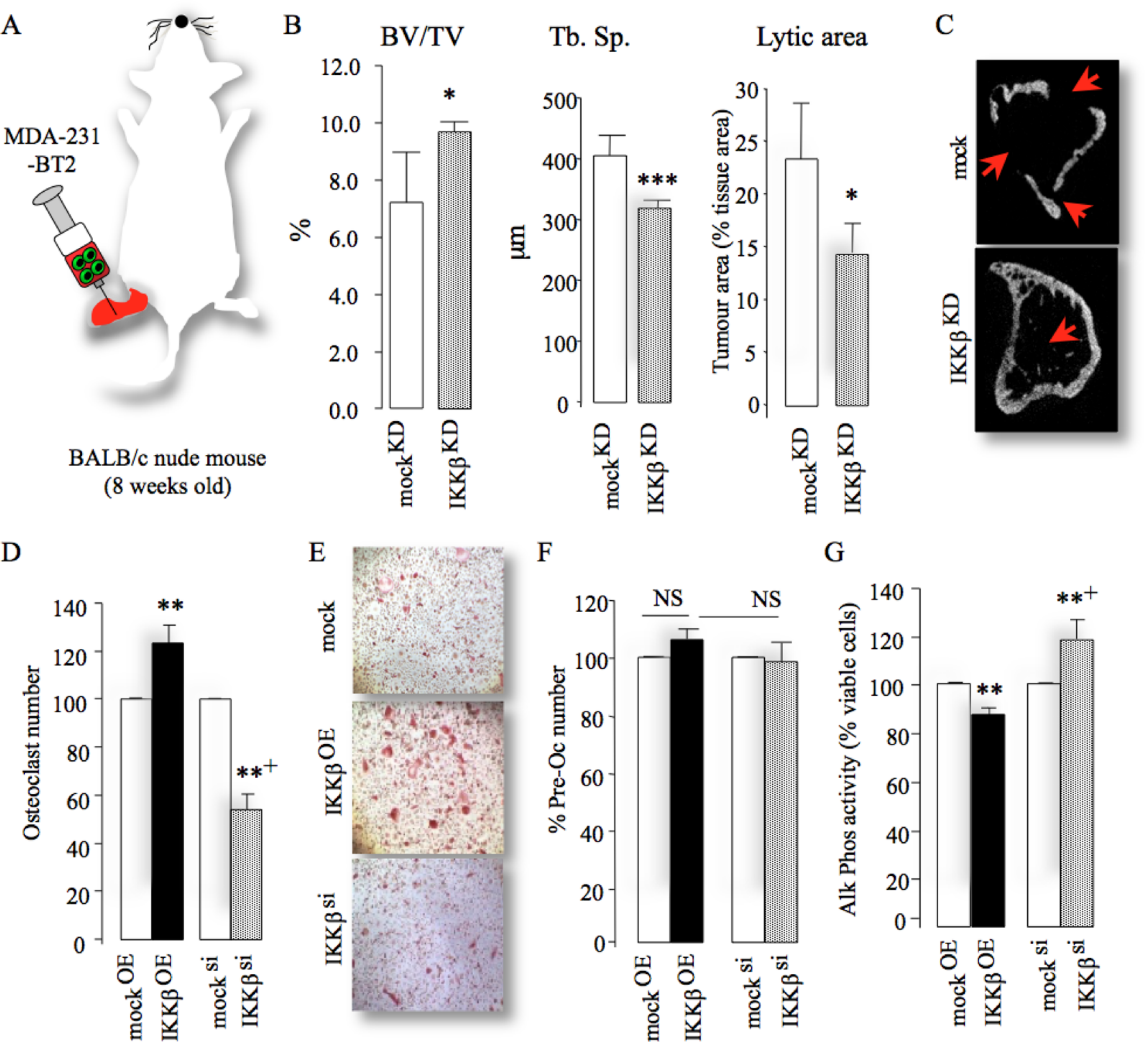
Cancer-specific IKKβ enhances local osteolysis (**A**) Graphic representation of intra-tibial injection of the osteotropic MDA-231-BT2 (MDA-BT2, *n* = 7 per group, 14 days) deficient in IKKβ (IKKβ^KD^) or their control (mock^KD^) in adult BALB/c -nu/nu athymic mice. (**B**) Bone volume (BV/TV, %, left), trabecular separation (Tb.Sp., middle) and lytic area (% of tissue area, right) in tibial metaphysis of mice from the experiment described in A. (**C**) Representative photomicrographs of microCT scan of tibial metaphysis of mice from the experiment described in panels A–B. Arrowheads denote osteolysis bone damage. (**D**–**F**) *In vitro* osteoclast number (D), pre-osteoclast proliferation (F) and osteoblast differentiation (G) after exposure to conditioned medium from control (mock^si^), IKKβ deficient (IKKβ^si^) or over-expressing (IKKβ^OE^) osteotropic human breast cancer cells. Representative photomicrographs of osteoclasts from the experiment described are shown in panel E. Values are mean ± SD; ^*^p < 0.05; ^**^p < 0.01; ^***^p < 0.001 from mock; ^+^*p <* 0.01 from IKKβ^OE^ cultures.

Next, we investigated the effects of manipulation of IKKβ on the interactions between osteotropic breast cancer cells, osteoblasts and osteoclasts *in vivo*, *ex vivo* and *in vitro*. All mice that received intra-tibial injection of human MDA-231-BT2 breast cancer cells suffered from osteolysis and bone damage as assessed by micro-CT analysis (Figure 4A–4C). In contrast, intra-tibial injection of MDA-231-BT2 deficient in IKKβ (Supplementary Figure 1E) caused less bone damage evident by increased bone volume, reduced trabecular separation and lytic area (Figure 4A–4C). Functional *in vitro* experiments in osteoblast and osteoclast cultures showed that exposure to conditioned medium from osteotropic MDA-231-BT1 cells overexpressing IKKβ increased osteoclast number (Figure 4D–4E) and inhibited osteoblast differentiation (Figure 4G), whereas these effects were revered in cultures treated with conditioned medium from IKKβ deficient cells. Further analysis of MDA-231-BT2 motility (Supplementary Figure 2B–2D) and growth (data not shown) confirmed that IKKβ overexpression and exposure with the osteolytic factor RANKL were both effective in significantly enhancing the migration of MDA-231-BT2 cells, whereas knockdown and pharmacological inhibition of IKKβ activity in these cells were inhibitory. These results show that IKKβ plays an important role in the behaviour of osteotropic breast cancer cells in bone.

### Tumour-derived VEGF contributes to IKKβ-driven osteolysis

**Figure 5:**
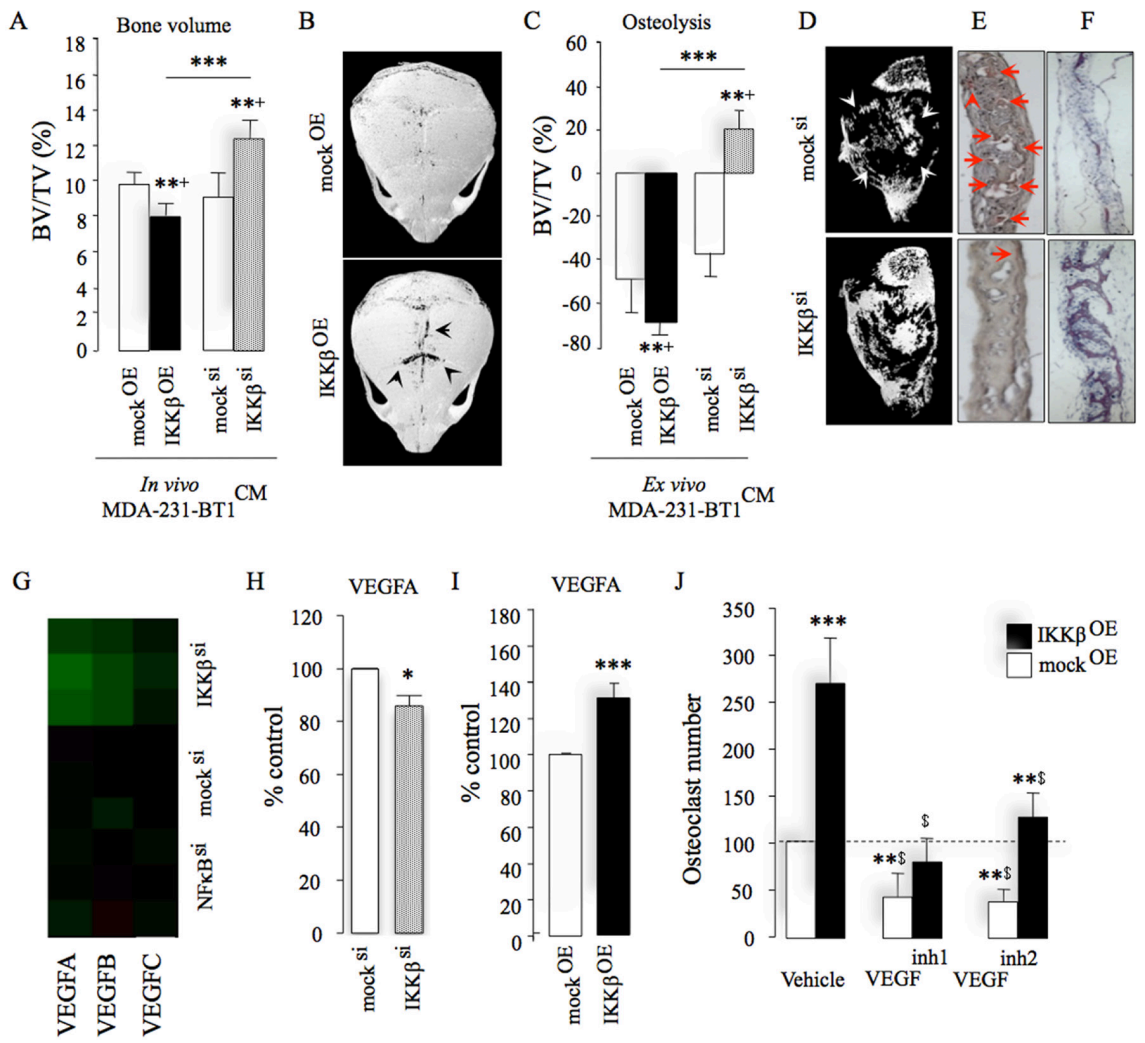
IKKβ regulates osteotropic breast cancer cell behaviour by engaging VEGFA (**A**) Total bone volume (BV/TV, %) in immunocompetent mice after supracalvarial injection of conditioned medium from control (mock^si/OE^), IKKβ deficient (IKKβ^si^) or over-expressing (IKKβ^OE^) osteotropic human MDA-231-BT1 breast cancer cells (*n* = 7). Representative photomicrographs of microCT scans of calvarial bone of mice from the experiment described in panel B. (**C**) *Ex vivo* osteolysis (BV/TV, %) in mouse calvaria organ culture treated with conditioned medium (20% v/v) from control (mock^si/OE^), IKKβ deficient (IKKβ^si^) or over-expressing (IKKβ^OE^) osteotropic human MDA-231-BT1 breast cancer cells (*n* = 7). (**D**–**F**) Representative photomicrographs of microCT scan of calvarial bone (D) from the experiment described in panel C and histological sections showing TRAcP positive osteoclasts (E) and bone (F) from the experiment described in panel A–B. Arrowheads in panel E denote osteoclasts. (**G**) Heat map of VEGFA, B and C genes showing the differential mRNA expression between control (mock^si^) or IKKβ deficient (IKKβ^si^) osteotropic human MDA-231-BT2 breast cancer cells. Green indicates repressed mRNA levels. (**H**) mRNA expression of VEGFA in control (mock^si^) or IKKβ deficient (IKKβ^si^) osteotropic human MDA-231-BT2 breast cancer cells. (**I**) Protein level of tumour-derived VEGFA in conditioned medium from control (mock^OE^) or over-expressing (IKKβ^OE^) osteotropic human MDA-231-BT2 breast cancer cells measured by human VEGFA ELISA kit. (**J**) *In vitro* osteoclast formation in M-CSF and RANKL stimulated bone marrow treated with conditioned medium (20% v/v) from control (mock^OE^) or over-expressing (IKKβ^OE^) osteotropic human MDA-231-BT2 breast cancer cells in the presence and absence of the selective VEGFR 1 inhibitors VEGF^inh1^ (ZM306416, 1 μM) or VEGF^inh2^ (Ki8751, 1 μM). Dotted line denotes osteoclast number in vehicle treated mock control. Values are mean ± SD; ^*^p < 0.05; ^**^p < 0.01; ^***^p < 0.001; ^$^*p <* 0.01 from vehicle treated mock (mock^KD^) control.

To investigate the mechanisms by which cancer-specific expression of IKKβ influence bone cell activity and osteolysis, we assessed conditioned medium induced osteolysis *ex vivo* and in immunocompetent adult mice (Figure 5A–5B). This experiment revealed that conditioned medium from MDA-231-BT1 cells overexpressing IKKβ caused more osteolytic bone damage in mouse calvarial bone *in vivo* (Figure 5A–5B) and *ex vivo* (Figure 5C) when compared to control cells (*p <* 0.01). Conversely, calvarial bone exposed to conditioned medium from IKKβ deficient MDA-231-BT1 cells suffered less bone damage *in vivo* (Figure 5A–5B) and *ex vivo* (Figure 5C) (*p <* 0.01). Further detailed histological analysis of the calvarial bone from the *ex vivo* experiment described in Figure 5C showed that mice injected with conditioned medium from IKKβ deficient MDA-231-BT1 cells exhibited less osteoclasts (47%, *p <* 0.05, figure not shown) and higher bone volume (40%, *p <* 0.05, figure not shown) when compared to mice injected with conditioned medium from mock control cells. Representative photomicrographs of histological sections showing osteoclasts and osteolysis are shown in Figure 5, panels E and F, respectively. Similar effects were also obtained in *ex vivo* experiments using calvarial organ culture exposed to conditioned medium from osteotropic human MDA-231-BT1 cells (Figure 5C) and mouse 4T1 breast cancer cells (data not shown). Encouraged by these findings, we employed an analytic approach that utilizes data from protein and gene expression arrays to attempt to identify the tumour-derived factor(s) responsible for the *in vivo* effects we observed. Analysis of the levels of human cytokines and chemokines in conditioned medium from mock and MDA-231-BT1 overexpressing IKKβ together with examination of the transcriptional responsiveness of these cells to changes in expression of IKKβ revealed that of all previously identified secreted bone metastasis factors [24, 25], only the expression of VEGF was significantly down regulated in response to IKKβ deficiency (*p <* 0.05, Figure 5G–5H). Analysis of a cohort of breast cancer patients with bone metastasis revealed that the expression of type A VEGF, but not B and C, is associated with disease occurrence (Supplementary Figure 3A). Moreover, VEGFA level was significantly elevated in conditioned medium from MDA-231-BT1 cells overexpressing IKKβ (Figure 5I) and mRNA expression was reduced in IKKβ deficient MDA-231-BT2 cells (Figure 5H). Consistent with this, the ability of MDA-231-BT1 overexpressing IKKβ cells to enhance RANKL-stimulated osteoclast formation was significantly reduced by antagonists of the VEGF receptor 1 and 2 when compared to mock control (Figure 5J), further confirming the involvement of VEGFA in IKKβ-driven osteotropic effects in the models described.

### IKKβ engages FoxO3A in osteotropic breast cancer cells

**Figure 6:**
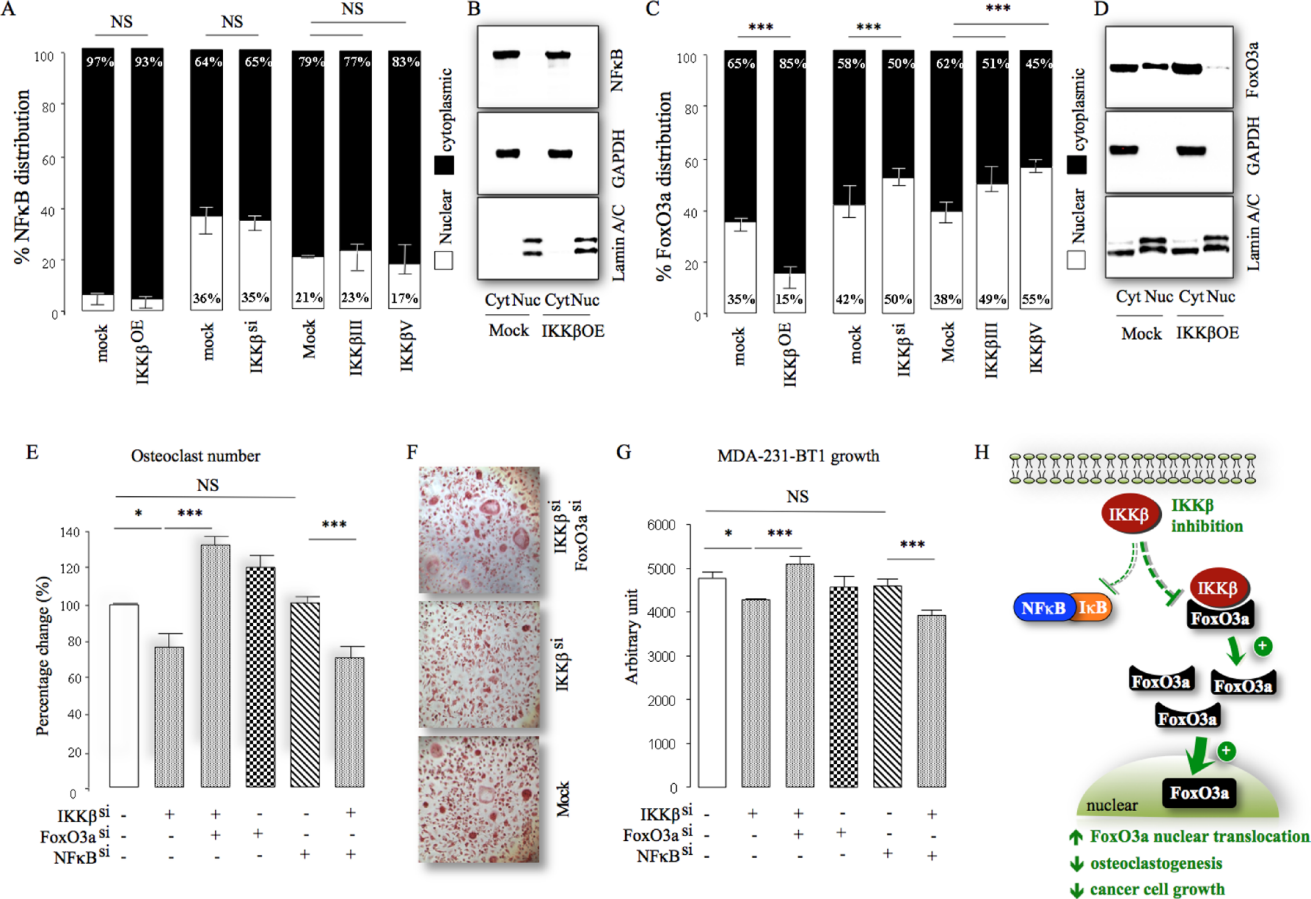
IKKβ engages FoxO3a in osteotropic breast cancer cells (**A**) Differential expression of p65NFκB in cytoplasm (Cyt) and nuclear (Nuc) fraction obtained from control (mock^si/OE^), IKKβ deficient (IKKβ^si^) or over-expressing (IKKβ^OE^) osteotropic human MDA-231-BT2 breast cancer cells cultured in the presence and absence of the selective IKKβ inhibitors IKKβIII or IKKβV (10 μM). (**B**) Representative photomicrographs of the expression of p65NFκB, GAPDH and Lamin A/C from the experiment described in panel A. (**C**) Differential expression of FoxO3a in cytoplasm (Cyt) and nuclear (Nuc) fraction obtained from control (mock^si/OE^), IKKβ deficient (IKKβ^si^) or over-expressing (IKKβ^OE^) osteotropic human MDA-231-BT2 breast cancer cells cultured in the presence and absence of the selective IKKβ inhibitors IKKβIII or IKKβV (10 μM). (**D**) Representative photomicrographs of the expression of FoxO3a, GAPDH and Lamin A/C from the experiment described in panel C. (**E**) *In vitro* osteoclastogenesis of murine M-CSF and RANKL stimulated bone marrow after exposure to conditioned medium (10% v/v) from control (mock^si/OE^), IKKβ deficient (IKKβ^si^), FoxO3a deficient (FoxO3a^si^), p65NFκB deficient (NFκB^si^) osteotropic human MDA-231-BT2 breast cancer cells. (**F**) Representative images of TRAcP positive osteoclasts from the experiment described in panel E. (**G**) *In vitro* viability of control (mock^si/OE^), IKKβ deficient (IKKβ^si^), FoxO3a deficient (FoxO3a^si^), p65NFκB deficient (NFκB^si^) osteotropic human MDA-231-BT2 breast cancer cells. (**H**) Schematic model for the proposed mechanism of IKKβ regulation of FoxO3a and NFκB nuclear translocation in osteotropic breast cancer cells. Dotted red lines denote inhibition, green arrows denote activation and upward and downward black arrows denote increase and decrease, respectively. Values are mean ± SD; ^*^p < 0.05; ^***^p < 0.001; NS, non-significant.

During the course of this investigation, we observed that knockdown, overexpression and pharmacological inhibition of IKKβ in the osteotropic MDA-231-BT2 had no significant effect on NFκB cytoplasmic retention and nuclear translocation (Figure 6A and 6B), despite significant changes of IKKβ kinase activity as measured by IκB phosphorylation (Supplementary Figure 1E) and degradation (Supplementary Figure 1F; knockdown - IκB phosphorylation - 78% reduction and IκB expression 126% increase, *p* < 0.01). In contrast, both knockdown and pharmacological inhibition of IKKβ in these cells significantly reduced FoxO3a cytoplasmic sequestering and enhanced FoxO3a nuclear localisation, whereas IKKβ overexpression was inhibitory (20% increase in cytoplasmic retention, *p <* 0.01) (Figure 6C–6D). We also found that the expression of the pro-apoptotic protein FoxO3a is significantly reduced in patients with metastatic breast cancer (*p* = 0.0028, *n* = 185, Supplementary Figure 3B). Functional studies in osteoclasts and MDA-231-BT1 cell cultures demonstrated that knockdown of FoxO3a expression, but not p65NFκB, restored the ability of MDA-231-BT2 deficient in IKKβ to enhance osteoclast formation (Figure 6E). FoxO3a knockdown protected MDA-231-BT2 against the growth inhibition caused by IKKβ inhibition (Figure 6G). Together, these results implicate the IKKβ/FoxO3a axis in the regulation of osteotropic breast cancer cell behaviour (Figure 6H).

## DISCUSSION

The IKK/NFκB signalling pathway plays a role in the development of breast cancer [17, 21], and inflammation-induced bone loss [7–10, 22]. Previous studies implicated both canonical and non-canonical NFκB activation in the regulation of breast cancer bone metastasis [10], and previous work by our laboratories has found that pharmacological inhibition of these pathways reduces the progression of breast cancer osteolytic metastatic [22]. However, the role of IKKβ, a key component of the canonical NFκB pathway, in the regulation of breast cancer bone metastasis remains poorly understood. Here, we report that IKKβ expression in osteolytic lesions is higher than in primary tumours obtained from breast cancer patients, and we describe a previously unknown role of cancer-specific IKKβ in the regulation of bone metastasis, skeletal tumour burden, osteolysis and bone cell activity associated with breast cancer. Whilst overexpression of IKKβ in the parental MDA-231 human breast cancer cells have failed to convey bone metastatic potential to these cells, it significantly enhanced the osteolytic abilities of osteotropic human MDA-231 breast cancer cells *in vitro*, *ex vivo* and *in vivo*. Furthermore, IKKβ overexpression significantly enhanced skeletal tumour burden and increased the incidence of bone metastasis, exacerbated osteolysis, worsened cachexia and increased mortality in mice injected with two independent osteotropic sub-clones of MDA-231 cells with different metastatic abilities. Conversely, knockdown of IKKβ in the aggressive osteotropic sub-clone of MDA-231 cells reduced their ability to cause osteolysis in both immuno-deficient and wild type mice. Although the effect of IKKβ knockdown/overexpression on breast cancer bone metastasis that we observed in this study were limited to the osteotropic MDA-231 breast cancer cells used, it is tempting to speculate that over-expressing IKKβ would likely have increased the ability of parental MDA-231 to metastasise to bone after intra-cardiac injection in mice. When combined with previous studies on the role of IKKβ in primary breast tumour development [17, 21] and bone remodelling [8], the present findings suggest that IKKβ expression by both primary and osteotropic breast cancer cells plays an essential role in breast cancer.

Breast cancer cells in the skeleton (osteotropic) acquire the capability to promote osteolysis by influencing the differentiation of osteoclasts and osteoblasts [4, 5, 27]. We, and others, have previously reported that pharmacological inhibition of NFκB activity reduced bone loss through inhibition of osteoclast bone resorption and stimulation of osteoblast differentiation [7–9, 11, 12]. Our current data expand on these observations and demonstrate that selective inhibition of IKKβ in osteotropic breast cancer cells suppressed their ability to enhance osteoclast formation and to inhibit osteoblast differentiation.

Mediators originating from skeletal tumours play a critical role in the regulation of the behaviour of breast cancer cells in bone [6, 28–30]. The NFκB signalling pathway is a well-established regulator of genes encoding various pro-inflammatory tumour-derived factors that are known to stimulate osteoclastogenesis, inhibit osteoblast differentiation, enhance angiogenesis and encourage tumour growth [17–21, 31]. Examination of the transcriptional responsiveness of osteotropic sub-lines of the breast cancer cell line MDA-231 to IKKβ inhibition and overexpression revealed that VEGF was the only tumour-derived factor that was affected by manipulation of IKKβ expression in the osteotropic MDA-231 cells used. VEGFA is a potent stimulator of angiogenesis and is known to enhance the growth and motility of triple negative cells via NFκB activation [32, 33]. In the present study, we detected elevated level of VEGFA in conditioned medium from two clones of osteotropic MDA-231 cells overexpressing IKKβ consistent of the high rate of angiogenesis and tumour cell proliferation in tumours observed *in vivo*. VEGF is also known to enhance osteoclast formation [34, 35] and consistent with this we have found that blockage of VEGF receptor in pre-osteoclasts abolished the ability of human and mouse osteotropic breast cancer cells to enhance osteoclastogenesis and to migrate. We speculate that the likely mechanism for the osteoblast inhibition that was observed following exposure to conditioned medium from IKKβ overexpression cells is likely to be due to a relative increase in VEGFA production since VEGF is known to suppress the maturation and activity of cells of the osteoblastic lineage [35, 36]. In contrast, we found no evidence to suggest that IKKβ in human osteotropic breast cancer cells or in osteoblasts regulate the expression of Parathyroid hormone-related protein (PTHrP), tumour necrosis factor alpha (TNFα), transforming growth factor beta (TGFβ), and Interleukin 6 (IL6) (data not shown), which all have been previously implicated in the regulation bone cell differentiation and activity [1, 2]. Notwithstanding this, we cannot exclude the indirect involvement of these essential mediators of osteolysis since VEGF and other tumour-derived factors are known to directly regulate their levels in osteoblast and osteoclasts [1, 2].

A major consequence of IKKβ activation is NFκB nuclear translocation and DNA binding [6, 37]. A surprising finding was that knockdown, overexpression and pharmacological inhibition of IKKβ failed to affect NFκB activation in the osteotropic MDA-231 breast cancer cells used. Our interpretation of these results is that tumour-derived factors in the conditioned medium – other than RANKL - activate NFκB via non-canonical and alternative pathways [6, 37–41]. During the course of this investigation, we became aware of studies that have reported that IKKβ binds, phosphorylates, and inhibits the pro-apoptotic transcription factor FoxO3a nuclear translocation [15, 21]. In agreement with the previously established role of FoxO3a in tumour growth [21, 42–46], osteoclast function and osteoblast differentiation [47], we have found that inhibition of IKKβ interaction with FoxO3a in osteotropic MDA-231 breast cancer cells overexpressing IKKβ inhibited their growth and diminished their ability to stimulate osteoclasts and to suppress osteoblast differentiation. In contrast, knockdown of p65NFκB in these cells had no effects. Whilst it is important to note that these findings do not exclude NFκB contribution to primary tumour growth, RANKL-induced osteoclast formation and osteoblast inhibition that have previously reported [48–51], our present data provide a plausible mechanism for the anti-migratory, anti-resorptive and osteoanabolic effects associated with IKKβ inhibition in the sub-clones of the osteotropic MDA-231 breast cancer cells used in this study.

Collectively, the results of the present study demonstrate that cancer-specific expression of IKKβ plays an essential role in the regulation of bone metastasis, skeletal tumour growth, osteolysis and enhanced bone cell activity associated with advanced breast cancer. These results offer new insight into the crosstalk between breast cancer cells and bone cells of the tumour microenvironment, and provide evidence to show that disruption of IKKβ and its interaction with FoxO3a may have potential therapeutic efficacy in all stages of breast cancer.

## MATERIALS AND METHODS

### Reagents and cultures

The parental human breast carcinoma cell lines MDA-MB-231 (MDA-231) and 4T1 originated from the American Type Culture Collection and were in culture for < 6 months. The osteotropic sub-clones of the human MDA-231 (MDA-231-BT1 and MDA-231-BT2) and mouse 4T1 (4T1-BT) cell-lines were generated by repeated passages *in vivo* [26]. The selective IKKβ inhibitors IKKβIII (401480) and IKKβV (401482) were purchased from CalBiochem (Dorset, UK); ZM306416 (2499) or Ki8751 (2542) were purchased from Tocris Bioscience (Bristol, UK). All solvents and reagents were purchased from Sigma-Aldrich (Dorset, UK) unless otherwise stated. Tissue culture medium was obtained from Invitrogen (Paisley, UK). Human macrophage colony stimulating factor (M-CSF, 416-ML-050) was purchased from R&D Systems (Abingdon, UK). Receptor activator of NFκB ligand (RANKL) was a gift from Patrick Mollat (Galapagos, France). The targeting IKKβ and non-targeting control siRNA were purchased from Thermo-Dharmacon (CO, USA). The antibodies anti-IKKβ, IKKα, IKKγ, pIκB (ser32), IκB, FoxO3a, GAPDH and Lamin A/C were purchased from Cell Signalling Technology (Boston, MA, USA) and NFκBp65 was purchased from Santa Cruz Biotechnology (Santa Cruz, CA, USA).

### Animal studies

All procedures involving mice and their care were approved by and performed in compliance with the guidelines of Institutional Animal Care and Use Committee of University of Edinburgh (Scotland, UK) and University of L’Aquila (L’Aquila, Italy) and conducted in conformity with national and international laws and policies (UK Home Office regulations; Italian Legislative Decree 116/9 and, Gazzetta Ufficiale della Repubblica Italiana no. 40, February 18, 1992;). C57BL/6J and BALB/c nu/nu mice were obtained from Harlan (UK) and Charles River (Milan, Italy), respectively.

### Orthotopic injection of tumour cells

Four week-old female BALB/c-*nu/nu* athymic mice were anesthetized with intraperitoneal injections of pentobarbital (60 mg/kg). Cells (1 × 10^6^ /50 μL PBS) were injected into the fat pad of the inguinal left breast (8 mice per group). Animals were daily monitored for cachexia (evaluated by body weight waste), behavior, and survival. The size of tumour was measured externally at least twice a week using a caliper and the formula of the volume of an ellipse [4/3 × π × (a × b × c)] was applied. Mice were euthanized by carbon dioxide inhalation after 55 days from tumour-cell injection. The primary tumours and the lungs of the animals were dissected and processed as described in [26].

### Intracardiac injection of tumour cells

Four week-old female BALB/c-nu/nu athymic mice were anesthetized and cancer cells (1 × 10^5^ /100 μL PBS) were injected into the left ventricle (16 mice per group) [26]. The development of metastasis in bone was monitored by X-ray (36 KPV for 10 seconds, Faxitron model n.43855A; Faxitron X-Ray Corp., Buffalo Grove, IL, USA) and *in vivo* micro-computed tomography analysis (microCT; Skyscan 1172 scanner, Skyscan, Belgium) [26]. Radiographs were scanned using the Bio-Rad scanning densitometer (Hercules, CA, USA). Mice were euthanized and subjected to microCT analysis and to anatomical dissection for evaluation of bone and visceral metastases.

### Intratibial injection of tumour cells

Seven four week-old female BALB/c-nu/nu athymic mice received intra-tibial injection cancer cells (5 × 10^4^ cells/ 20μL PBS) in the left leg [26]. Animals were euthanized 14 days (4T1) or 21 days (MDA-231) post injection and bones were analyzed by micro–computed tomography (MicroCT, Skyscan 1172 scanner). Skeletal tumor growth was measured on 2D microCT images using Image J (1.34s; NIH, Bethesda, MD, USA).

### Supracalvarial injection of conditioned medium

Three weeks-old wild type female C57BL/6J mice were injected over the calvarial bones with conditioned medium from cancer cells on 7 consecutive days. Mice were scarified 3 days after the last injection and bone density was assessed using microCT at a resolution of 8 mm. No mice in this study exhibited any obvious physical signs of illness or inflammatory response.

### *Ex vivo* experiments

Neonatal mouse calvarias were isolated from 7 day-old mice, incubated in standard alpha-MEM for 24 hour and divided into equal halves along the median sagittal suture. Excised calvarial half was placed into culture on stainless steel rafts in 48-well plates in the presence and absence of cancer cells (1 × 10^4^ cells/well). Tissue culture medium was changed every 48h hours and fresh control medium or medium containing test agents was added and the cultures were terminated after 7 days. For studies involving conditioned medium, conditioned medium form breast cancer cells (20% v/v) was prepared and added to the calvarial organ cultures. Calvarial bone density was assessed using microCT at a resolution of 5 mm.

### Tumour histology, immunohistochemistry and histomorphometry

Orthotopic tumours were excised, fixed and embedded in paraffin. Sections were cut (5 μm) using a Reichert-Jung 1150/Autocut microtome, incubated with antibodies and visualised using the Ultra-Vision Detection System anti-Polyvalent HRP/DAB kit according to the manufacturer’s instructions. Metastatic bones were embedded in methyl-methacrylate without decalcification. Von gieson-Von Kossa staining was performed to evaluate the tumour burden, Hematoxilin and Eosin staining for evaluating osteoclast parameters and toluidine blue staining for osteoblast. Histomorphometric measurements were carried out using an interactive image analysis system (IAS 2000, Delta Sistemi, Rome, Italy).

Paraffin embedded material from human breast cancer and matched bone metastases were retrieved from the archive of Pathology Department of San Salvatore Hospital (L’Aquila, Italy). Histological sections were deparaffinized, incubated with 0.07 M citrate buffer (pH 6), 15 min at 98° C, for antigen retrieval, treated with 3% H_2_O_2_ and incubated for 40 min at room temperature with the anti-IKKβ rabbit polyclonal antibody (Catalogue number: LS-B1362, LifeSpan BioSciences, Inc., Seattle, WA). The staining signals were revealed using the Dako LSAB+ System-HRP. Slides were counterstained with Mayer’s hematoxylin. Human placenta was used as positive control whereas negative controls were performed in parallel by omitting primary antibody for each case. IKKβ expression was evaluated in invasive primary carcinoma, *in situ* carcinoma (where present) and bone metastases and recorded as percentage of positively stained cells and staining intensity (%IKKβ, 0–40% is defined as week, 40–60 is defined as moderate and 60–100% is defined as high).

### Small RNA interference

Cancer cells were transfected with siRNA (100 nM, Dharmafect 1 reagent, Dharmacon, CO, USA) according to the manufacturer’s instructions. Small interfering RNAs (siRNA; siGenome SMART pool) as a pool of four annealed double-stranded RNA oligonucleotides for IKKβ (M-003503-03) and their non-targeting control no. 3 (D001201-03) (Dharmacon, CO, USA) were used. The efficiency of knockdown was determined by Western blot analysis.

### Generation of stable cell lines

To generate IKKβ expressing retroviruses 293T packaging cells (1 × 10^5^ cell/cm^2^) were transfected with the 5 μg pMX retroviral vector (empty or containing IKKβ), 5 μg pCMV-Gag-pol vector, 5 μg pCMV-VSV-G vector and 40 μl polyethylenimine. pLKO.1 puro lentiviral plasmid empty (TRC Lentiviral pLKO.1 Empty Vector Control RHS4080) or containing IKKβ-shRNA (TRC Lentiviral Human IKBKB shRNA RHS4533-EG3551 glycerol set) were purchased from Thermo-Dharmacon (CO, USA). To generate IKKβ-shRNA expressing lentivirus 293T-packaging cells (1 × 10^5^ cell/cm^2^) were transfected with the 5 μg pLKO.1 lentiviral vector (empty or containing IKKβ), 5 μg psPAX2 vector, 5 μg pCMV-VSV-G vector and 40 μl polyethylenimine. Medium containing viral particles was collected, filtered applied immediately to MDA-231 cells, which had been plated 18 hours before infection at a density of 1 × 10^4^ cells/cm^2^. Polybrene (107689 Sigma-Aldrich Dorset, UK) was added to a final concentration of 5 mg/ml, and the retroviruses supernatants were incubated with the MDA-231 cells. Twenty-four hours after infections, cells were selected with 1mg/ml puromycin for 72 hours.

### Osteoblast cultures

Primary osteoblasts were isolated from the calvarial bones of 2-day-old mice by sequential collagenase digestion. For bone nodule assay, osteoblasts were seeded into 12-well plates (10 × 10^5^ cells/well) for up to 21 days in αMEM supplemented with 10% FCS, penicillin and streptomycin, β-glycerol phosphate (10 μM) and L-ascorbic acid (50 μg/ml). Osteoblast number, differentiation and bone nodule formation were determined by AlamarBlue assay, alkaline phosphatase (Alk Phos) assay and alizarin red (ALZ) staining, respectively. Activity of Alk Phos was assessed by a colorimetric assay, which measures the conversion of p-nitrophenyl phosphate to p-nitrophenyl in cell lysate and activity was normalized to cell number as determined by the AlamarBlue assay as previously described [52].

### Osteoclast cultures

Osteoclast formation, survival and activity were studied using mouse RANKL and M-CSF generated mouse osteoclasts. Bone marrow (BM) cells were flushed from the long bones of 3–5 month old mice and were incubated for 48 hours in αMEM supplemented with mouse M-CSF (100 ng/ml). For osteoclast generation, M-CSF-generated bone marrow macrophages (osteoclast precursor cells) were cultured in αMEM supplemented with M-CSF (25 ng/ml) and RANKL (100 ng/ml). For studies involving conditioned medium, conditioned medium form cancer cultures was prepared in αMEM and added to osteoclast cultures (10% v/v). Osteoclasts were identified using TRAcP staining and manually counted on a Zeiss Axiovert light microscope.

### Assessment of cell motility

The migration of cancer cells was assessed by wound healing assay [22]. Briefly, confluent cell layers were scored with a fine pipette tip and migration of cells was monitored using an Olympus ScanR time lapse microscope system. Percentage of wound closure were analysed using Tscratch analysis program.

### Western blotting

Cell monolayer was gently scraped in standard lysis buffer (0.1% (w/v) SDS, 0.5% (w/v) sodium deoxycholate, 1% Triton X-100, 1 μM EDTA, 2% (v/v) protease inhibitor cocktail, 10 mM of sodium fluoride and 2% (v/v) phosphatase inhibitor cocktail. For studies involving Cytoplasmic/Nuclear fractions, cell monolayer was scraped in Cytoplasmic lysis buffer (10 mM Tris [pH 7.5], 0.05% NP-40, 3 mM MgCl2, 100 mM NaCl, 1 mM EGTA) supplemented with protease and phosphatase inhibitor cocktail. Cell pellets were then re-suspended in complete RIPA lysis buffer and protein concentration was measured using BCA assay (Pierce, USA). Total protein (70 mg) was resolved by SDS-PAGE on 12% polyacrylamide SDS gels, transferred onto PVDF membranes (BioRAD, UK) and immunoblotted with appropriate antibodies. The immuno-complexes were visualised by an enhanced chemiluminescence detection kit (Pierce, USA) using horseradish peroxidase-conjugated secondary antibody (Jackson labs, UK), and then visualised using chemiluminescence (Amersham, UK) on a Syngene GeneGnome imaging system.

### Measurement of levels of tumour-derived factors

Levels of vascular endothelial growth factor (VEGF) in MDA-231 and 4T1 conditioned medium was determined by enzyme linked immunosorbent assay (ELISA) (R&D Systems, Abingdon, UK) and Proteome Profiler Human XL Cytokine Array Kit (ARY022, R&D Systems, Abingdon, UK), according to the manufacturer’s instructions.

### Measurement of NFκB activity

Nuclear extracts from bone and cancer cells were prepared using a nuclear extract kit (Active Motif, Rixensart, Belgium) and DNA binding was measured using TRANSAM ELISA kit for p65 NFκB (Active Motif, Rixensart, Belgium), according to the manufacturer’s instructions.

### RNA extraction and microarray

Ribonucleic acid (RNA) was extracted using RNeasy Mini Kit with RNAse-free DNAse treatment (74014, Qiagen Crawley, United Kingdom), according the manufacturer’s instructions. 500 ng of total RNA from each sample was amplified and biotinylated using Illumina^®^ TotalPrep RNA Amplification Kit (Ambion, AMIL1791), according the manufacturer’s instructions, and then quantified using a Bioanalyser 2100. 750 ng of high-quality labeled cRNA per sample was hybridized to Illumina HumanHT-12v3 Expression Beadarrays in triplicate (Illumina, Cambridge, United Kingdom) using Whole-Genome Expression Direct Hybridisation kit (Illumina) and scanned with a 500GX (Illumina). The raw and normalized microarray data have been deposited in the Gene Expression Omnibus (GEO) database under accession number GSE71444.

### Analysis of clinical data

Retrospective analysis of IKKβ expression with respect to primary tumour growth and bone relapse was conducted using published cohorts of data. The MSK82, EMC192 and EMC286 datasets were directly integrated with batch-correction using ComBat. Exhaustive survival analysis was performed by considering all possible cut-points using the survivALL package. *P* values were derived from Wilcoxon test and were two-tailed. Immunohistochemical analysis of IKKβ was also performed in cohort of 13 primary breast carcinomas and 5 matched bone metastasis samples obtained from the Pathology Unit, San Salvatore hospital (L’Aquila, Italy), with informed consent from all subjects.

### Statistical analysis

Results were reported as mean ± standard deviation (SD) as indicated in figure legends. A *p*-value value of 0.05 or below was considered statistically significant. The half maximal inhibitory concentration (IC50) values were calculated using GraphPad Prism 4.0 for windows. Other comparisons were performed using unpaired two-sided Student’s *t* test or analysis of variance (ANOVA) followed by Dunnet’s post test using SPSS for Windows version 11. Statistical analysis of the microarray data was performed using R and BioConductor packages. Quantile normalisation was performed using the *lumi* package. Comparisons between Kaplan-Meier curves were performed using the a log-rank (Mantel-Cox) test. Meta-analysis was performed using Review Manager (RevMan), version 5.3 (Review Manager, 2014).

## SUPPLEMENTARY MATERIALS FIGURES AND TABLES



## Abbreviations

NFκB: nuclear factor kappa-B
FoxO3a: Forkhead box O3A
IKK: IκB kinase
RANK: receptor activator of NFκB
RANKL: RANK ligand
M-CSF: mouse macrophage colony stimulating factor
DMSO: Dimethyl sulfoxide
microCT: micro–computed tomography
TRAcP: tartrate-resistant acid phosphatase
Alk Phos: alkaline phosphatase
ALZ: alizarin red
BM: bone marrow
ANOVA: analysis of variance
SD: standard deviations
iRNA: Small interfering RNA
VEGF: vascular endothelial growth factor
ELISA: enzyme linked immunosorbent assay
IC50: half maximal inhibitory concentration
TNFα: tumour necrosis factor alpha
IL6: interleukin 6
TGFβ: transforming growth factor beta
PTHrP: Parathyroid hormone-related protein

## References

[1] Mundy GR (2002). Metastasis to bone: causes, consequences and therapeutic opportunities. Nat Rev Cancer.

[2] Roodman GD (2004). Mechanisms of bone metastasis. N Engl J Med.

[3] Coleman RE (2001). Metastatic bone disease: clinical features, pathophysiology and treatment strategies. Cancer Treat Rev.

[4] Yoneda T, Hiraga T (2005). Crosstalk between cancer cells and bone microenvironment in bone metastasis. Biochem Biophys Res Commun.

[5] Zhang XH, Jin X, Malladi S, Zou Y, Wen YH, Brogi E, Smid M, Foekens JA, Massagué J (2013). Selection of bone metastasis seeds by mesenchymal signals in the primary tumor stroma. Cell.

[6] Karin M (2008). The IkappaB kinase-a bridge between inflammation and cancer. Cell Res.

[7] Ruocco MG, Maeda S, Park JM, Lawrence T, Hsu LC, Cao Y, Schett G, Wagner EF, Karin M (2005). I{kappa}B kinase (IKK){beta}, but not IKK{alpha}, is a critical mediator of osteoclast survival and is required for inflammation-induced bone loss. J Exp Med.

[8] Idris AI, Krishnan M, Simic P, Landao-Bassonga E, Mollat P, Vukicevic S, Ralston SH (2010). Small molecule inhibitors of IkappaB kinase signaling inhibit osteoclast formation *in vitro* and prevent ovariectomy-induced bone loss *in vivo*. FASEB J.

[9] Otero JE, Dai S, Alhawagri MA, Darwech I, Abu-Amer Y (2010). IKKbeta activation is sufficient for RANK-independent osteoclast differentiation and osteolysis. J Bone Miner Res.

[10] Park BK, Zhang H, Zeng Q, Dai J, Keller ET, Giordano T, Gu K, Shah V, Pei L, Zarbo RJ, McCauley L, Shi S, Chen S, Wang CY (2007). NF-kappaB in breast cancer cells promotes osteolytic bone metastasis by inducing osteoclastogenesis via GM-CSF. Nat Med.

[11] Chang J, Wang Z, Tang E, Fan Z, McCauley L, Franceschi R, Guan K, Krebsbach PH, Wang CY (2009). Inhibition of osteoblastic bone formation by nuclear factor-kappaB. Nat Med.

[12] Alles N, Soysa NS, Hayashi J, Khan M, Shimoda A, Shimokawa H, Ritzeler O, Akiyoshi K, Aoki K, Ohya K (2010). Suppression of NF-kappaB increases bone formation and ameliorates osteopenia in ovariectomized mice. Endocrinology.

[13] Crawford A, Nahta R (2011). Targeting Bcl-2 in Herceptin-Resistant Breast Cancer Cell Lines. Curr Pharmacogenomics Person Med.

[14] Wu L, Shao L, An N, Wang J, Pazhanisamy S, Feng W, Hauer-Jensen M, Miyamoto S, Zhou D (2011). regulates the repair of DNA double-strand breaks induced by ionizing radiation in MCF-7 breast cancer cells. PLoS One.

[15] Tezil T, Bodur C, Kutuk O, Basaga H (2012). IKK-β mediates chemoresistance by sequestering FOXO3; a critical factor for cell survival and death. Cell Signal.

[16] Oida K, Matsuda A, Jung K, Xia Y, Jang H, Amagai Y, Ahn G, Nishikawa S, Ishizaka S, Jensen-Jarolim E, Matsuda H, Tanaka A (2014). Nuclear factor-ĸB plays a critical role in both intrinsic and acquired resistance against endocrine therapy in human breast cancer cells. Sci Rep.

[17] Lee DF, Kuo HP, Chen CT, Hsu JM, Chou CK, Wei Y, Sun HL, Li LY, Ping B, Huang WC, He X, Hung JY, Lai CC (2007). IKK beta suppression of TSC1 links inflammation and tumor angiogenesis via the mTOR pathway. Cell.

[18] Yeh PY, Lu YS, Ou DL, Cheng AL (2011). IκB kinases increase Myc protein stability and enhance progression of breast cancer cells. Mol Cancer.

[19] Bist P, Leow SC, Phua QH, Shu S, Zhuang Q, Loh WT, Nguyen TH, Zhou JB, Hooi SC, Lim LH (2011). Annexin-1 interacts with NEMO and RIP1 to constitutively activate IKK complex and NF-κB: implication in breast cancer metastasis. Oncogene.

[20] Neil JR, Schiemann WP (2008). Altered TAB1: I kappaB kinase interaction promotes transforming growth factor beta-mediated nuclear factor-kappaB activation during breast cancer progression. Cancer Res.

[21] Hu MC, Lee DF, Xia W, Golfman LS, Ou-Yang F, Yang JY, Zou Y, Bao S, Hanada N, Saso H, Kobayashi R, Hung MC (2004). IkappaB kinase promotes tumorigenesis through inhibition of forkhead FOXO3a. Cell.

[22] Idris AI, Libouban H, Nyangoga H, Landao-Bassonga E, Chappard D, Ralston SH (2009). Pharmacologic inhibitors of IkappaB kinase suppress growth and migration of mammary carcinosarcoma cells *in vitro* and prevent osteolytic bone metastasis *in vivo*. Mol Cancer Ther.

[23] Chen DT, Nasir A, Culhane A, Venkataramu C, Fulp W, Rubio R, Wang T, Agrawal D, McCarthy SM, Gruidl M, Bloom G, Anderson T, White J (2010). Proliferative genes dominate malignancy-risk gene signature in histologically-normal breast tissue. Breast Cancer Res Treat.

[24] Kang Y, Siegel PM, Shu W, Drobnjak M, Kakonen SM, Cordón-Cardo C, Guise TA, Massagué J (2003). A multigenic program mediating breast cancer metastasis to bone. Cancer Cell.

[25] Blanco MA, LeRoy G, Khan Z, Alečković M, Zee BM, Garcia BA, Kang Y (2012). Global secretome analysis identifies novel mediators of bone metastasis. Cell Res.

[26] Rucci N, Capulli M, Ventura L, Angelucci A, Peruzzi B, Tillgren V, Muraca M, Heinegard D, Teti A (2013). Proline/arginine-rich end leucine-rich repeat protein N-terminus is a novel osteoclast antagonist that counteracts bone loss. J Bone Miner Res.

[27] Siclari VA, Guise TA, Chirgwin JM (2006). Molecular interactions between breast cancer cells and the bone microenvironment drive skeletal metastases. Cancer Metastasis Rev.

[28] Yamamoto Y, Gaynor RB (2001). Therapeutic potential of inhibition of the NF-kappaB pathway in the treatment of inflammation and cancer. J Clin Invest.

[29] Coussens LM, Werb Z (2002). Inflammation and cancer. Nature.

[30] Abu-Amer Y (2009). Inflammation, cancer, and bone loss. Curr Opin Pharmacol.

[31] Huang HL, Chiang CH, Hung WC, Hou MF (2015). Targeting of TGF-β-activated protein kinase 1 inhibits chemokine (C-C motif) receptor 7 expression, tumor growth and metastasis in breast cancer. Oncotarget.

[32] Di Benedetto M, Toullec A, Buteau-Lozano H, Abdelkarim M, Vacher S, Velasco G, Christofari M, Pocard M, Bieche I, Perrot-Applanat M (2015). MDA-MB-231 breast cancer cells overexpressing single VEGF isoforms display distinct colonisation characteristics. Br J Cancer.

[33] Shibata A, Nagaya T, Imai T, Funahashi H, Nakao A, Seo H (2002). Inhibition of NF-kappaB activity decreases the VEGF mRNA expression in MDA-MB-231 breast cancer cells. Breast Cancer Res Treat.

[34] Isowa S, Shimo T, Ibaragi S, Kurio N, Okui T, Matsubara K, Hassan NM, Kishimoto K, Sasaki A (2010). PTHrP regulates angiogenesis and bone resorption via VEGF expression. Anticancer Res.

[35] Hu K, Olsen BR (2016). Osteoblast-derived VEGF regulates osteoblast differentiation and bone formation during bone repair. J Clin Invest.

[36] Deckers MM, Karperien M, van der Bent C, Yamashita T, Papapoulos SE, Löwik CW (2000). Expression of vascular endothelial growth factors and their receptors during osteoblast differentiation. Endocrinology.

[37] Clément JF, Meloche S, Servant MJ (2008). The IKK-related kinases: from innate immunity to oncogenesis. Cell Res.

[38] Tanaka T, Shibazaki A, Ono R, Kaisho T (2014). HSP70 mediates degradation of the p65 subunit of nuclear factor κB to inhibit inflammatory signaling. Sci Signal.

[39] Pazarentzos E, Mahul-Mellier AL, Datler C, Chaisaklert W, Hwang MS, Kroon J, Qize D, Osborne F, Al-Rubaish A, Al-Ali A, Mazarakis ND, Aboagye EO, Grimm S (2014). IκΒα inhibits apoptosis at the outer mitochondrial membrane independently of NF-κB retention. EMBO J.

[40] Yan J, Xiang J, Lin Y, Ma J, Zhang J, Zhang H, Sun J, Danial NN, Liu J, Lin A (2013). Inactivation of BAD by IKK inhibits TNFα-induced apoptosis independently of NF-κB activation. Cell.

[41] Dondelinger Y, Jouan-Lanhouet S, Divert T, Theatre E, Bertin J, Gough PJ, Giansanti P, Heck AJ, Dejardin E, Vandenabeele P, Bertrand MJ (2015). NF-κB-Independent Role of IKKα/IKKβ in Preventing RIPK1 Kinase-Dependent Apoptotic and Necroptotic Cell Death during TNF Signaling. Mol Cell.

[42] Chen J, Gomes AR, Monteiro LJ, Wong SY, Wu LH, Ng TT, Karadedou CT, Millour J, Ip YC, Cheung YN, Sunters A, Chan KY, Lam EW, Khoo US (2010). Constitutively nuclear FOXO3a localization predicts poor survival and promotes Akt phosphorylation in breast cancer. PLoS One.

[43] Karadedou CT, Gomes AR, Chen J, Petkovic M, Ho KK, Zwolinska AK, Feltes A, Wong SY, Chan KY, Cheung YN, Tsang JW, Brosens JJ, Khoo US, Lam EW (2012). FOXO3a represses VEGF expression through FOXM1-dependent and -independent mechanisms in breast cancer. Oncogene.

[44] Storz P, Döppler H, Copland JA, Simpson KJ, Toker A (2009). FOXO3a promotes tumor cell invasion through the induction of matrix metalloproteinases. Mol Cell Biol.

[45] Yang JY, Zong CS, Xia W, Yamaguchi H, Ding Q, Xie X, Lang JY, Lai CC, Chang CJ, Huang WC, Huang H, Kuo HP, Lee DF (2008). ERK promotes tumorigenesis by inhibiting FOXO3a via MDM2-mediated degradation. Nat Cell Biol.

[46] Zou Y, Tsai WB, Cheng CJ, Hsu C, Chung YM, Li PC, Lin SH, Hu MC (2008). Forkhead box transcription factor FOXO3a suppresses estrogen-dependent breast cancer cell proliferation and tumorigenesis. Breast Cancer Res.

[47] Kawamura N, Kugimiya F, Oshima Y, Ohba S, Ikeda T, Saito T, Shinoda Y, Kawasaki Y, Ogata N, Hoshi K, Akiyama T, Chen WS, Hay N (2007). Akt1 in osteoblasts and osteoclasts controls bone remodeling. PLoS One.

[48] Liu M, Sakamaki T, Casimiro MC, Willmarth NE, Quong AA, Ju X, Ojeifo J, Jiao X, Yeow WS, Katiyar S, Shirley LA, Joyce D, Lisanti MP (2010). The canonical NF-kappaB pathway governs mammary tumorigenesis in transgenic mice and tumor stem cell expansion. Cancer Res.

[49] Cao Y, Karin M (2003). NF-kappaB in mammary gland development and breast cancer. J Mammary Gland Biol Neoplasia.

[50] Cao Y, Luo JL, Karin M (2007). IkappaB kinase alpha kinase activity is required for self-renewal of ErbB2/Her2-transformed mammary tumor-initiating cells. Proc Natl Acad Sci USA.

[51] Yamaguchi N, Ito T, Azuma S, Ito E, Honma R, Yanagisawa Y, Nishikawa A, Kawamura M, Imai J, Watanabe S, Semba K, Inoue J (2009). Constitutive activation of nuclear factor-kappaB is preferentially involved in the proliferation of basal-like subtype breast cancer cell lines. Cancer Sci.

[52] Idris AI, Rojas J, Greig IR, Van’t Hof RJ, Ralston SH (2008). Aminobisphosphonates cause osteoblast apoptosis and inhibit bone nodule formation *in vitro*. Calcif Tissue Int.

