# Supplementary material for: Regulation of breast cancer induced bone disease by cancer-specific IKKβ
Source: Oncotarget. 2018 Mar 23;9(22):16134–48. doi: 10.18632/oncotarget.24743 (PMC5882323; doi:10.18632/oncotarget.24743)
Supplement: Supplementary file 1 [file oncotarget-09-16134-s001.pdf]

Regulation of breast cancer induced bone disease by cancer-specific IKKβ

SUPPLEMENTARY MATERIALS

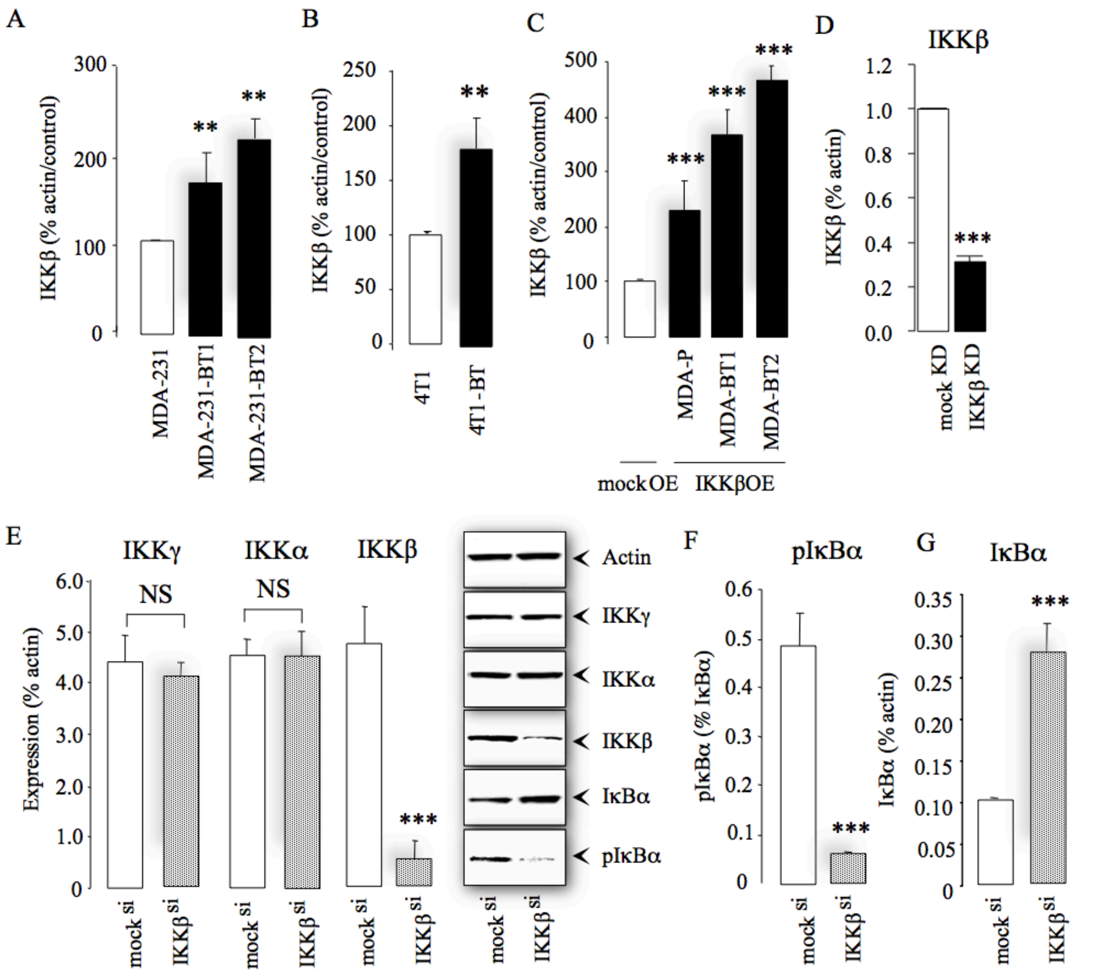

Supplementary Figure 1: IKKβ expression and activity in parental and osteotropic human breast cancer cells (related to Figures 1 to 5). (**A**) Differential expression of IKKβ in the mildly (MDA-231-BT1) and highly metastatic (MDA-231-BT2) clones of the parental human MDA-231 breast cancer cells. (**B**) Differential expression of IKKβ in the metastatic and osteotropic (4T1-BT) clone of the parental mouse 4T1 breast cancer cells (4T1). (**C**) Differential expression of IKKβ in total cell lysates obtained from control (mock^{OE}) and over-expressing (IKKβ^{OE}) human breast cancer cells. (**D**) Differential expression of IKKβ and actin in total cell lysates obtained from mock control (mock^{KD}) and IKKβ deficient (IKKβ^{KD}) MDA-MB-BT2 human breast cancer cells. (**E**) Differential expression of IKKγ, α, β and actin in total cell lysates obtained from mock control or MDA-MB-BT1-IKKβ^{si} human breast cancer cells. (**F–G**) Differential expression of phosphorylated and total IκBα and actin in total cell lysates obtained from (mock^{si}) or IKKβ deficient (IKKβ^{si}) osteotropic human MDA-231-BT1 breast cancer cells. Values are mean ± SD; $^{**}p < 0.01$; $^{***}p < 0.001$.

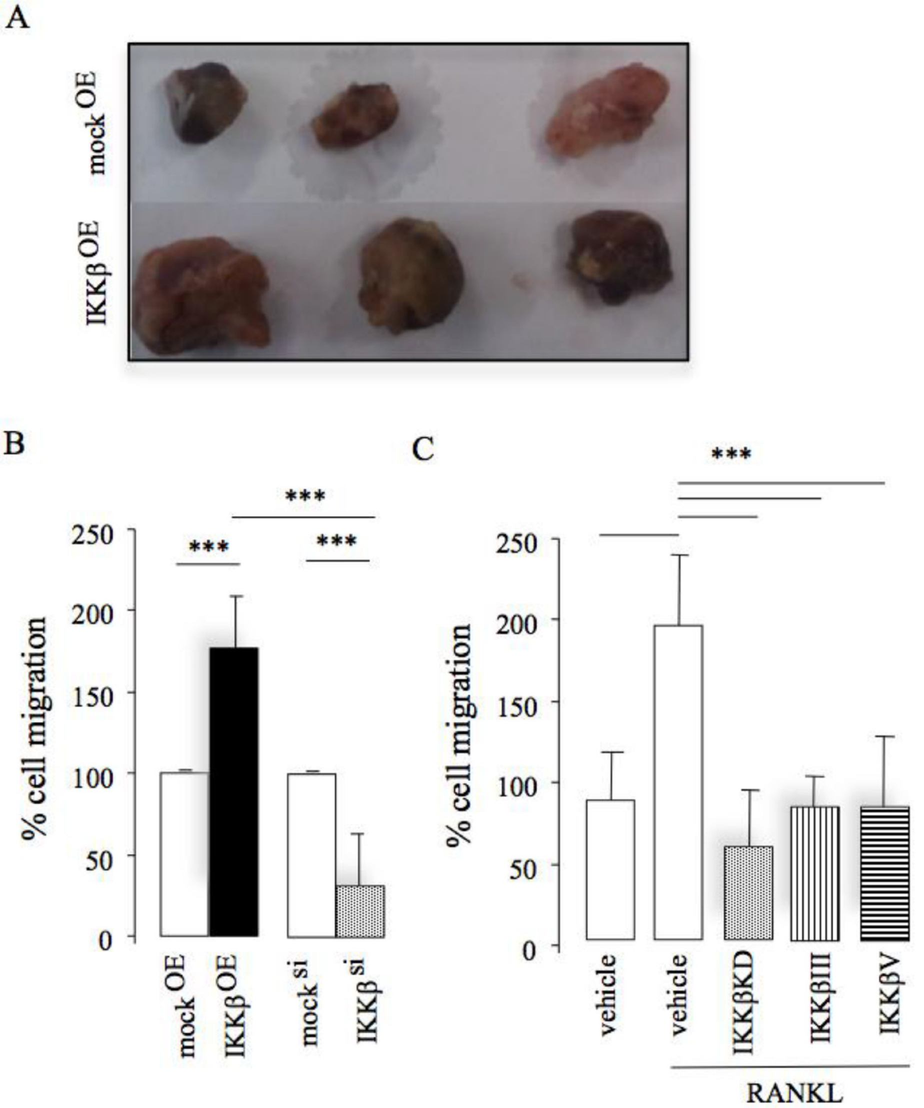

Supplementary Figure 2: Effects of IKKβ manipulation on tumour cell growth and motility (related to Figure 2). (**A**) Representative images of tumours from 6 mice from the experiment described in Figure 2 panels (A–B). (**B**) *In vitro* 2D directed migration of mock control ($\text{mock}^{OE/si}$), IKKβ over-expressing ($\text{IKK}\beta^{OE}$) and IKKβ deficient ($\text{IKK}\beta^{si}$) MDA-231-BT2 human breast cancer cells. (**C**) *In vitro* 2D directed migration of RANKL (100 ng/ml) stimulated mock and IKKβ deficient MDA-231-BT2 cells in the presence and absence of the selective IKKβ inhibitors IKKβIII or IKKβV (10 µM). Values are mean ± SD; $^{**}p < 0.01$; $^{***}p < 0.001$. At least two different shRNAs were tested in *in vitro* experiments in which IKKβ deficient cells were used (data not shown).

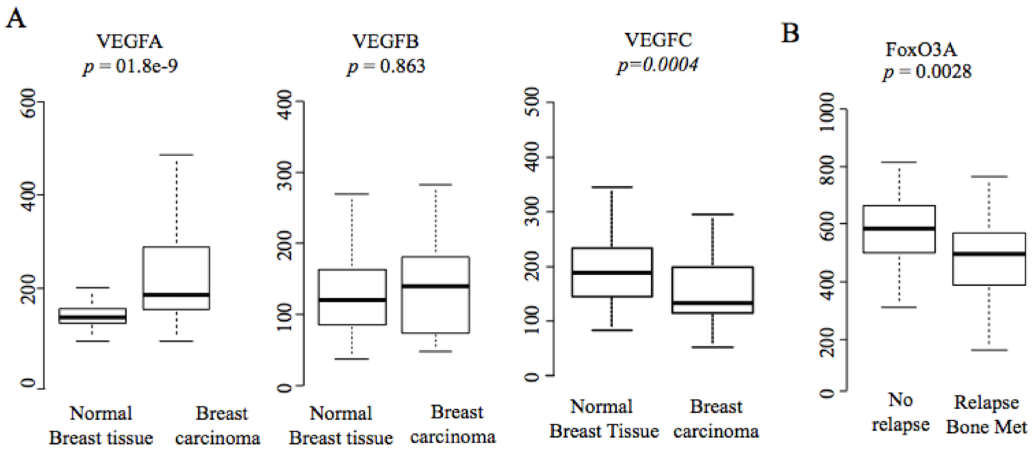

Supplementary Figure 3: (*Related to Figures 5 and 6*). (**A**) Increased expression of VEGFA, but not VEGFB or VEGFC, is associated with breast cancer recurrence in patients (n = 560). (**B**) Retrospective analysis of breast tissue samples (n = 185) showing reduced expression of FoxO3a is associated with relapse in breast cancer patients.
